# Supplementary material for: Change in plasma cryptococcal antigen titers in Uganda among outpatients with advanced HIV disease from 2017 to 2022 with rollout of national cryptococcal screening
Source: PLoS One. 2026 Jun 2;21(6):e0349854. doi: 10.1371/journal.pone.0349854 (PMC13229320; doi:10.1371/journal.pone.0349854)
Supplement: Attachment — Submitted filename: PONE-D-25-65883 R1 Comments to authors.docx [file pone.0349854.s001.docx]

Plos Submission: PONE-D-25-65883

General comments:

This is a well written article that generates important information on CrAg titers in a Sub-Saharan setting. It may be outside the scope of this study, but it would be valuable to add how many CrAg positive participants received antifungal treatment and a lumbar puncture.

Comments to authors:

Major issues

In the Introduction, please provide some perspective of the ART coverage for Uganda for the study period. This would be important as the data seems to indicate increasing ART coverage among PLHIV. This could then be contrasted to the lower ART coverage for the CrAg positive participants.

Table 1 is confusing as it is not clear whether the median CD4 count or the number of participants are reported by year. It would be better to split this into two tables, one with frequencies and a second with the median and IQR values.

It would be valuable to add a decision tree to this manuscript to graphically depict the number of CrAg positive (436), how many are on ART, have meningitis symptoms, etc. Is there any data as well on whether antifungal treatment was administered, which is a major gap in our data systems. Would it also be possible to add how many had a CD4 ≤200 cells/µL that were CrAg negative as well.

In Results, please indicate how many PLHIV were CrAg negative. In addition, the CrAg detection rate would be important for comparison purposes to other settings.

Figure 1 appears to report data by quarters for the period between 2019 and 2022. The legend refers to quartiles. Please report all four quarters for each year from 2017 to 2022. If this data is not available, perhaps report the data by year.

Minor issues

In the abstract, please indicate that the CD4 threshold was a count ≤200 cells/µL. Please also indicate that advanced HIV disease in this study excludes clinical staging (I to IV).

The abstract would read better if sub-sections were added for Introduction, Methods, Results and Conclusions.

In the Background on line 54, please indicate the CD4 threshold for the CrAg screening program. Please also indicate whether this was a reflexed of provider-initiated approach.

In the Methods in line 69 – CrAg is repeated in the same sentence.

Line 85 – Please revise RStudio 2024 to ‘RStudio: Integrated Development Environment for R’
