# Supplementary material for: Change in plasma cryptococcal antigen titers in Uganda among outpatients with advanced HIV disease from 2017 to 2022 with rollout of national cryptococcal screening
Source: PLoS One. 2026 Jun 2;21(6):e0349854. doi: 10.1371/journal.pone.0349854 (PMC13229320; doi:10.1371/journal.pone.0349854)
Supplement: Attachment — Submitted filename: LetterofResponse_PLoSOne_4May2026.doc [file pone.0349854.s004.doc]

November 30, 2026

Manuscript: "Change in plasma cryptococcal antigen titers in Uganda among outpatients with advanced HIV disease from 2017 to 2022 with rollout of national cryptococcal screening" PONE-D-25-65883

Dear Dr. Bongomin,

Thank you for your further detailed review of our manuscript, “Change in plasma cryptococcal antigen titers in Uganda among outpatients with advanced HIV disease from 2017 to 2022 with rollout of national cryptococcal screening”. We have gone through the editor’s and reviewers’ comments in detail and have addressed all the editorial suggestions.

Below is our point-by-point reply to the reviewers’ comments in italics.

**Reviewer #2:** All aspects raised have been addressed in the revision of this manuscript. It would be interesting to receive a subsequent analysis that includes additional outcomes such as the lumbar puncture data.

*Response: Thank you for your comments and suggestions, we will consider this for future projects.*

**Reviewer #3:** Overall, this is improved, clearer, and the authors addressed the earlier reviewer comments well. Below are a few minor comments to tighten methods clarity and interpretation.

1. Abstract, Methods (lines 30–31) “We prospectively screened adults with advanced HIV disease (CD4<200 cells/μL) for CrAg from 2017 through 2022 using the lateral flow assay and assessed median plasma CrAg titer.”
- It would be helpful to specify whether participants with prior cryptococcal meningitis were excluded, both in the Abstract and main Methods.

*Response: Thank you for your comment. We did not exclude people with a prior history of cryptococcal meningitis, as CrAg screening is routine. In this manuscript we summarize CrAg screening and titers by month and quarter. The inclusion criteria for the study is: adults with advanced HIV disease who received a positive CrAg plasma result with an associated titer performed (see lines 89-91). Given that we haven’t specified anything about prior cryptococcal meningitis, the reader can assume that we did not exclude participants with prior cryptococcal meningitis.*

2. Abstract, Results (line 32) “From November 2017 to May 2022, 436 adults with advanced HIV disease had a positive plasma CrAg test.”
- Apologies if this was discussed already in the prior review, but is there a denominator and percent positive?

*Response: No, we cannot present a denominator. We are not reporting prevalence of CrAg positivity here. We are reporting trends in titer over time. We do not have data on all CrAg negative individuals (who were thereby ineligible for our study).*

3. Lines 39–40: “Despite expanded access to ART nationally, 58% of study participants in 2022 were ART-naïve, suggesting ongoing late presentation to care for people with advanced HIV disease.”
- This is reasonable interpretation, but ART-experienced patients who still develop cryptococcosis may be an equally important signal of poor adherence, treatment interruption, virologic failure, or incomplete immune recovery. May consider broadening the interpretation to include both late presentation and ineffective long-term treatment or retention.

*Response: Thank you, this is an interesting point to consider, and we have added context to the manuscript regarding the implication of participants that are not ART-naïve presenting to care with a low CD4 count and positive CrAg result.*

*Please see lines 45-46 of the abstract:*

*“In addition, the presence of ART-experienced patients in our cohort suggests challenges with treatment adherence and retention.”*

*And, lines 169-186 and 269-271 in the discussion:*

*“Additionally, the presence of participants in our cohort that were ART-experienced and yet still presented with a CD4 count less than 200 and were CrAg-positive suggests challenges with treatment adherence, interruption of treatment, virologic failure, or incomplete immune recovery.”*

*“In addition, the presence of ART-experienced patients in our study population suggests ongoing challenges with treatment adherence and retention.”*

4. Methods (lines 80–82) “Participants in this analysis were adults with advanced HIV disease who received a CrAg-positive result in plasma with an associated titer performed.”
- Were individuals with a history of CM excluded?

*Response: No, individuals with a history of CM were not excluded if they met the inclusion criteria listed. We have not specifically mentioned this, because it was not an exclusion criterion.*

5. The Table presents variables that are not described in the Methods. If accurate, state that age, sex, CD4, ART status, time on ART, symptoms, and titer were collected and analyzed. Would be helpful to specify which meningitis symptoms were assessed.

*Response Thank you for your comment. We have specified the symptoms that were part of the assessment in the table caption:*

*“Symptoms of meningitis included one or more of the following: headache, nuchal rigidity, photophobia, confusion, focal neurologic deficits, mania, or fever.” – lines 139-141*

*Additionally, we have added the following sentence (lines 97-98) to the Methods:*

*“Baseline characteristics including age, sex, CD4 count, ART status, time on ART, and symptoms at presentation were collected and analyzed.”*

6. Discussion (lines 138–139) “We had hypothesized that the CrAg titer would decrease given the increased availability of ART and rollout of the national CrAg screening program in Uganda.”
- Another likely explanation may be that this is not just about ART access. It may also reflect adherence challenges, treatment interruption, stigma, retention failures, etc. If adherence data were not collected, the authors might consider acknowledging this as a limitation in interpreting the findings.

*Response: Yes, in our cohort, ART access was shown to be in decline, which we address in the discussion. See lines 164-168 of the discussion:*

*“Notably, in our cohort, 58% were ART naïve in both 2021 and 2022, the highest rates over our study period, suggesting ongoing late presentation to care. There was a statistically significant decline in prevalence of ART coverage at enrollment over time. Thus, there is a disconnect between the national reports of improvement in ART coverage, and ART coverage of this population with advanced HIV disease.”*

*Adherence data was collected, as we have data of participants that were currently on ART, never on ART, and previously on ART. See Table 1 on line 138.*

*We have added more context to the discussion regarding the implications of participants that are not ART-naïve presenting with a positive CrAg plasma result. Please see lines 169-186 and 269-271 in the discussion:*

*“Additionally, the presence of participants in our cohort that were ART-experienced and yet still presented with a CD4 count less than 200 and were CrAg-positive suggests challenges with treatment adherence, interruption of treatment, virologic failure, or incomplete immune recovery.”*

*“In addition, the presence of ART-experienced patients in our study population suggests ongoing challenges with treatment adherence and retention.”*

7. If screening volume or coverage increased over time, later years may reflect improved capture of high-risk patients rather than stable or worsening epidemiology alone. This may be hard to prove, but it may be a real potential bias that may be considered.

*Response: Thank you for this thoughtful comment. We have added this as a limitation in the Discussion (lines 225-227).*

*“If volume of screening changed over time, trends may reflect better documentation of titer or CrAg result, rather than worsening epidemiology”*

8. “Screening occurred at outpatient HIV clinics in the Kampala and Wakiso districts and the Masaka Medical Research Council field station…”
- Along the same lines, were these same catchment areas represented throughout the study period? If geographic coverage changed over time, that could alter case mix.

*Response: True. In our case, these same areas were represented throughout the study period.*

Reviewer #4: Change in plasma cryptococcal antigen titers in Uganda among outpatients with
advanced HIV disease from 2017 to 2022 with rollout of national cryptococcal
screening
This version is very much improved and I have only a few minor commets
Abstract
Page 2 Lines 38-39 : “Despite expanded access to ART nationally, 58% of study participants in 2022 were ART-naïve” ….These results are in the conclusion but are not in the results section of the abstract

*Response: Thank you, we have now added to the results section of the abstract, see lines 40-41.*

*“There was a statistically significant decline in the percentage of participants taking ART at the time of screening (p<0.001), with 58% reporting never having taken ART in 2022.”*

Introduction
Page 3 Lines 61: “Elevated CrAg titer indicates higher risk of meningitis and death’… This seems to be a repeat of Lines 58-59

*Response: Agreed, we have deleted this sentence in line 58-59.*

Pages 3-4 Lines 66-68: “We hypothesize that CrAg titer has decreased annually, as people are presenting to care earlier in their disease course before onset of fulminant meningitis”. However, the subjects enrolled in the study were either newly enrolled into care, returning to care, or had ceased taking ART. For the hypothesis to be valid, should the study patients be only those who are newly enrolled to care? Those participants returning to care, or had ceased taking ART are repeat participants to care

*Response: Thank you for this thoughtful comment. Our study population includes those with advanced HIV. The proportion newly engaged in care vs. returning to care is unknown, and quite difficult to discern. Our hypothesis was that the expansion of ART availability and the national CrAg screening program would lead to presentation to care earlier in the disease course. This might be true for ART naïve individuals, but also for ART experienced individuals. Perhaps with expanded access to ART, people who are ART-experienced are less likely to stay out of care for so long. Or are more likely to resume care and get CrAg screened.*

Results
A number of studies in Uganda indicate that men tend to present later for HIV care than women however in this study, the number of males and females were almost equal. Any reason for this?

Men’s late presentation for HIV care in Eastern Uganda: The role of masculinity norms | PLOS One

*Response: This is a good question. While men may present to HIV care later and with more advanced disease, they will eventually reach care in similar numbers. This may be a reason why males and females are represented in equal numbers in our study. It is possible that men presented with more advanced HIV disease. While interesting, that is not the purpose of this study, so we have not pursued this further.*

All authors have approved of the manuscript. The manuscript is not under consideration elsewhere and has not been previously published. There are no conflicts of interest by any author.

Warmly,

Radha Rajasingham, MD

Associate Professor

Infectious Diseases & International Medicine

Department of Medicine, University of Minnesota
